# The relationship between dyslipidemia and inflammation among adults in east coast China: A cross-sectional study

**DOI:** 10.3389/fimmu.2022.937201

**Published:** 2022-08-11

**Authors:** Najiao Hong, Yongjun Lin, Zhirong Ye, Chunbaixue Yang, Yulong Huang, Qi Duan, Sixin Xie

**Affiliations:** ^1^ Department of General Medicine, The First Hospital of Quanzhou affiliated to Fujian Medical University, Quanzhou, China; ^2^ Department of Mathematics, Virginia Tech, Blacksburg, VA, United States

**Keywords:** dyslipidemia, IL-6, TNF-a, MCP-1, TBARS, TAC

## Abstract

**Objective:**

Dyslipidemia is one of the major public health problems in China. It is characterized by multisystem dysregulation and inflammation, and oxidant/antioxidant balance has been suggested as an important factor for its initiation and progression. The objective of this study was to determine the relationship between prevalence of dyslipidemia and measured changes in the levels of proinflammatory cytokines (IL-6, TNF-a, and MCP-1), thiobarbituric acid-reactant substances (TBARS), and serum total antioxidant capacity (TAC) in serum samples.

**Study design:**

A cross-sectional survey with a purposive sampling of 2,631 enrolled participants (age 18–85 years) was performed using the adult population of long-term residents of the municipality of east coast China in Fujian province between the years 2017 and 2019. Information on general health status, dyslipidemia prevalence, and selected mediators of inflammation was collected through a two-stage probability sampling design according to socioeconomic level, sex, and age.

**Methods:**

The lipid profile was conducted by measuring the levels of total cholesterol (TC), high-density lipoprotein cholesterol (HDL-C), low-density lipoprotein cholesterol (LDL-C), and triglycerides (TG) with an autoanalyzer. Dyslipidemia was defined according to National Cholesterol Education Program Adult Treatment Panel III diagnostic criteria, and patients with it were identified by means of a computerized database. Serum parameters including IL-6/TNF-a/MCP-1, TBARS, and TAC were measured in three consecutive years. Familial history, education level, risk factors, etc. were determined. The association between dyslipidemia and serum parameters was explored using multivariable logistic regression models. Sociodemographic, age, and risk factors were also investigated among all participants.

**Results:**

The mean prevalence of various dyslipidemia in the population at baseline (2017) was as follows: dyslipidemias, 28.50%; hypercholesterolemia, 26.33%; high LDL-C, 26.10%; low HDL-C, 24.44%; and hypertriglyceridemia, 27.77%. A significant effect of aging was found among all male and female participants. The mean levels of serum Il-6/TNF-a/MCP-1 were significantly higher in all the types of dyslipidemia among male participants. Female participants with all types of dyslipidemia but low HDL-C showed an elevation of IL-6 and MCP-1 levels, and those with dyslipidemias and hypercholesterolemia presented higher levels of TNF-a compared to the normal participants. The oxidative stress marker TBARS increased among all types of dyslipidemia except hypertriglyceridemia. All participants with different types of dyslipidemia had a lower total antioxidant capacity. Correlation analysis showed that cytokines and TBARS were positively associated with age, obesity, and diabetes mellitus, but not sex, sedentary leisure lifestyle, hypertension, and CVD/CHD history. The activity of TAC was negatively associated with the above parameters.

**Conclusions:**

The correlation between the prevalence of dyslipidemia and the modification of inflammation status was statistically significant. The levels of proinflammatory cytokines, oxidative stress, and antioxidant capacity in serum may reflect the severity of the lipid abnormalities. These promising results further warrant a thorough medical screening in enhanced anti-inflammatory and reduced oxidative stress to better diagnose and comprehensively treat dyslipidemia at an early stage.

## Introduction

Dyslipidemia is recognized worldwide as one important risk factor in cardiovascular disease, morbidity, and mortality. Some studies suggested an independent correlation between dyslipidemia and the risk of cardiovascular and encephalovascular events (1, 2). It is characterized by a systemic abnormal lipid profile in which the level of serum cholesterol, triglycerides, or both is elevated, or the level of high-density lipoprotein cholesterol (HDL-C) is reduced (1, 2).

The occurrences and developments of some lipid disorders are often associated with excessive induction of proinflammatory cytokine production. Previous studies showed that cytokines such as TNF-α were involved in the severity of the lipid disturbance. Certain proinflammatory cytokines not only are involved in regulating energy balance, proliferation, and apoptosis of adipocytes, but also regulate lipolysis, inhibit lipid synthesis, and lower blood lipids, among others. Many reports indicated that proinflammatory cytokines may significantly influence the development of lipid metabolism of obesity, atherosclerosis, steatohepatitis, hyperlipoproteinemia, and type 2 diabetes (3, 4). The study by Scicali et al. (5) observed the biomarker role of S100A12, a molecule expressed primarily by neutrophils in familial hypercholesterolemia (FH) in which the level of serum S100A12 was correlated not only with age and genetic mutation but also with pulse wave velocity. Another study (6) indicated that the ligation of S100A12 with its receptor-advanced glycation end products (RAGE) occurs downstream of activation of intracellular signal cascades, e.g., MAP-kinase and nuclear factor (NF)-κB, resulting in production of proinflammatory cytokines such as TNF-α and IL-1β, and high expression of adhesion molecules such as intercellular adhesion molecule (ICAM)-1 and vascular cell adhesion molecule (VCAM)-1. A recent report by Kim et al. (7) demonstrated a correlation between serum S100A12 levels and the vascular calcification score, suggesting the progression of atherosclerotic vascular complications consequent to the induction of systemic inflammation. A clinical study by Kroon et al. (8) indicated the evidence of an increased arterial wall inflammation in familial dysbetalipoproteinemia (FD) accompanied by elevated lipid accumulation in monocytes and higher expression of surface integrins (CD11b, CD11c, and CD18). Overall, these findings imply that inflammation is a key regulatory process that hitches the immune system and hypercholesterolemia, which contributes to the increased cardiovascular disease risk.

Several studies have shown an imbalance between oxidants and antioxidants in impaired lipid metabolism due to the altered lipid peroxidation and antioxidant enzyme activities (9–12). According to the hypothesis of allostasis by McEwen et al. (13), it is possible that prolonged oxidative stress, as the mediators of allostatic load, may induce a long-term alteration in lipid profile concomitant with altered antioxidant status, resulting in severe consequence such as atherosclerosis.

In the last two decades, many reports have shown the estimated prevalence of dyslipidemia with the following difference rates: 59.74% in the state of São Paulo of Brazil (2014) (14), 53% in U.S. (2006) (15), 88.9% in Thailand (16). 79.55% in Turkey (2014) (17), 79.00% in India (2014) (18), 56.50% in Japan (2015) (19), and 19.05% in China (2006) (20).

In China, the east coast has been undergoing rapid socioeconomic development and urbanization in the past four decades. In the 21st century, an increase in industrialization and an acceleration of rural-to-urban migration are occurring along east coast provinces, which are now confronted with a major public health challenge, especially the growing disease burden attributable to environmental conditions, food and nutrition, and lifestyle choices (21).

While China is known to have a rapidly increasing epidemiology of lipid metabolic dysfunction, there have been few effective studies on complete evaluation of proinflammation and oxidative stress to assess the magnitude of the dyslipidemia profile among the adults in east coast. The evidence of the prevalence of dyslipidemia and trends of proinflammatory markers and oxidative stress within a distinct group representative of east coast urban population will undoubtedly ensure proper guidelines of healthcare resources for both primary and secondary prevention of cardiovascular disease and atherosclerosis (22–26).

The present study aimed to determine a 3-year change in the levels of proinflammatory markers, oxidative stress, antioxidant capacity, and the prevalence of dyslipidemias from a large representative sample of the urban population ≥18 years old residing in east coast urban, Fujian province, China. The correlations among the serum markers, e.g., IL-6, TNF-α, MCP-1, TBARS, and TAC, and lipid disorders were explored as one of changing times and public health significance.

## Methods

### Study population and sample

Our cross-sectional study was carried out through a two-stage stratified sampling method in which a representative sample was selected among the general population aged ≥ 18 years in east coast, Fujian, China. According to the method by Yang et al. (26), the first level of sampling in the study population was stratified by indicated geographical regions of the east coast. The selected areas encompassed six urban districts—Fuzhou, Xiamen, Quanzhou, Zhangzhou, Putian, and Ningde. For the second stage of sampling, classified factors included sex, age distribution, education level, and socioeconomic strata based on Fujian urban districts data published in the year of 2016. According to Kish selection table (27) and the government (Fujian Provincial Bureau of Statistics) household registration profile, especially the data on the household and residential compositions, eligible households and its adult members were randomly chosen on each listed street/avenue/road of 89 units with probability proportional to population size. The representative participants were qualified from urban civilian adults who had been living in their current city for ≥5 years. Any participants who may not be available due to personal reasons were replaced by other eligible households within a similar local community during the recruiting time. In the second level of sampling, participants were stratified according to ages, sex, general health status, and educational status as well as social status.

Ethical approval and consent to all participants and the study was approved by the hospital Ethical Committee of Quanzhou in accordance with the Declaration of Helsinki. The health survey that the current study conducted was adopted by the Chinese Resident Health Literacy Scale (CRHLS), which was developed by the Chinese Ministry of Health. The scale’s reliability and construct validity were assessed by using a population-based sample from South China by Shen et al. (28) and others (29, 30). It was prepared in simplified Chinese and consisted of 64 selected items based on classical test theory and item response theory. A pre-test pertinent to field test, consultation process, and plain language examination was displayed in a pilot group recruited from five cities. It was noticeable that the test was time-consuming because it took 25–35 min for a participant to complete and even longer for those with limited literacy or other conditions. The scales showed not only a balance between complexity and quantity of questions in different categories of the questionnaire but also a good psychometric property with construct validity, reliability, and acceptability. The overall performance of existing CRHLS in the current study was a qualified tool in accuracy of predictive performance during the home interview. In detail, the participants needed to complete a survey questionnaire about demographic features, socioeconomic status, lifestyle, health/medical history especially hypertension, atherosclerosis, and diabetes, smoking, and alcohol consumption. Exclusion criteria included those who showed very low literacy or poor communication skills, those with severe organic or neurologic pathology, and those unable to process an interview due to psychiatric disorders. Participants who met the exclusion criteria should be replaced by other eligible participants recruited within a similar community. The criterion of smoking was defined as continuous use of tobacco or quit within 4 months. The criterion of drinking was defined as having 30 g/week of alcohol intake for the past 12 months or quit within 4 months. The physical activity levels were classified as regular physical activity and sedentary leisure based on the global physical activity questionnaire. Education levels were graded as lower or college/high school, and occupation was categorized as white-collar workers, blue-collar workers, and unemployed/unskilled/housewives. As suggested by Fujian Bureau of Statistics, the average annual income per capita of the participant’s household in five consecutive years (expressed in US dollars) was classified into three major categories: low (<$6,030), medium ($6,030–10,960), and high (>$10,960).

### Participant characteristics

**Table 1** shows the demographic and socioeconomic strata among the selected participants in six age groups compared to the whole east coast urban adults in Fujian province and the overall urban population of the province based on Fujian 2016 census data (31). Fujian province has a population of about 76 hundred million, out of which 38.1 million people live in six major cities along the east coast, namely, Fuzhou, Xiamen, Quanzhou, Zhangzhou, Putian, and Ningde.

**Table 1:** Distribution of demographic characteristics and general socioeconomic indicators among different urban populations.

Characteristic	The population in the current study			Fujian coast urban population	Fujian urban Population
	1st year (2017)	Follow-up I (2018)	Follow-up II (2019)		
Total number	15,397	12,144	11,762	9,936,850	3,495,356
Age distribution (years) (%)					
18–29	14.9	14.1	14.2	15.8	16.1
30–39	17.7	17.0	17.1	18.5	17.4
40–49	19.8	20.5	19.7	21.4	19.5
50–59	20.8	21.0	20.6	19.2	20.2
60–69	17.1	17.8	18.9	15.5	17.3
≥70	9.7	9.6	9.5	9.6	9.5
Median (years)	41.8	41.9	42.0	41.1	42.2
Sex					
Male	50.08	49.90	49.82	50.12	50.88
Female	49.92	50.10	50.18	49.88	49.12
Education					
Lower	53.02	52.90	51.91	53.89	65.26
College/high school	46.98	47.10	48.09	46.11	34.74
Occupation					
White-collar workers	34.1	33.9	33.1	34.0	32.6
Blue-collar workers	42.2	41.5	41.8	44.9	42.6
Unemployed, retired and housewives	23.7	24.6	25.1	21.1	24.8
Income					
Low	20.1	19.3	18.3	21.2	29.5
Medium	63.0	63.6	64.1	62.4	57.3
High	16.9	17.1	17.6	16.4	13.2
Marital status					
Married	73.1	72.5	70.9	74.3	74.7
Single	26.9	27.5	29.1	25.7	25.3

A total of 2,631 individuals (1,368 male and 1,266 female individuals) aged 18 to 85 years between 2017 and 2019 were selected before registration in the study. There was an overall response rate of 91.1% (2,397), 88.6% (1,209) among male adults and 93.8% (1,188) among female adults. In the year of the original investigation, 31 participants opted out of the study for various reasons, and the replacement rate was 1.3%. A total of 2,001 participants with an 83.46% overall response rate entered the first year of the follow-up study. Thirty (1.5%) participants had to discontinue their interest in the second year of follow-up. By the end of 2019, 1,962 individuals with 963 men and 999 women were invited to complete the investigation. During the three years, registered participants had three analyses in each calendar year.

### Collection of blood samples

As the second step, a series of clinical examinations were carried out: health-related questionnaire, blood pressure, fasting blood glucose, lipid profiles, proinflammatory markers, and oxidative stress.

The serum samples were later analyzed for serum lipids. Eighty milliliters of fasting blood was obtained after 12 h overnight fasting and kept for 30 min to allow the blood to clot. All the serum samples were separated and stored at –70°C. The biochemical analyses and methods were performed for the following parameters: glycemia by blood gas analyzer (Bayer 865, NY), and lipid including total cholesterol, serum triglycerides, and HDL-C by the enzymatic method [Hitachi 7600 auto-analyzer (Hitachi, Japan)].

### Lipid level analysis

Serum LDL-C was estimated by using the Friedewald formula: LDL-C = TC – HDL-C – TG/5. The value of non-HDL-C was calculated through the equation: non-HDL-C (mg/dl) = TC - HDL-C. It was suggested by NCEP-ATP III guidelines that dyslipidemia was characterized within four lipid components as hypercholesterolemia (TC ≥ 240 mg/dl) or hypertriglyceridemia (TG ≥ 160 mg/dl) or low HDL-C (≤40 mg/dl), or high LDL-C (≥160 mg/dl).

### Proinflammatory marker measurement

The levels of serum proinflammatory markers—interleukin-6, monocyte chemoattractant protein-1, and tumor necrosis factor-α—were detected by using enzyme-linked immunosorbent assay (ELISA) (MultiSciences Biotech Co., Ltd.) according to the manufacturer’s protocol. As suggested by the ELISA kit, a mixture of 50 μl of serum samples, twofold diluted standard, and diluted detection antibody were incubated at room temperature for 1 h and 45 min on a microplate shaker set at 300 rpm. After careful washing, 100 µl of diluted streptavidin-horseradish peroxidase was added. Then, progressive development of a colored complex in the plates was produced by using a substrate solution. The intensity of the produced colored complex was directly proportional to the concentration of the markers in the samples. The absorbance of the complex is finally measured and calculated by plotting against the expected concentration, forming a standard curve.

### Thiobarbituric acid reactive substance measurement

Determination of serum TBARS was tested by thiobarbituric acid assay according to the method by Kamal et al. (32). First, 50 μl of serum samples was purified with butylated hydroxytoluene in 10% trichloroacetic acid. Then, 0.6% thiobarbituric acid in 0.44 M phosphoric acid was added; after 45-min incubation at 90°C, the pink color complex was developed. Finally, the absorbance of the complex was measured and calculated by plotting against the 1,1,3,3 tetramethoxypropane standard curve.

### Total antioxidant capacity measurement

Total antioxidant capacity was assessed by a spectrophotometric method. First, the diluted serum sample [1:25 in phosphate buffer saline (pH = 7.4)] was mixed with 0.1 mM 2,2 diphenyl-1-picrylhydrazyl reagent. Then, the mixture was incubated in a dark room for 30 min. After centrifuging at 14,000 rpm, the absorbance was measured and finally calculated by plotting against the standard curve.

### Statistical analysis

Demographics, health-related and medical histories, social status, and biomedical parameters were tested by SPSS 21.0 software (SPSS, Inc.). Means and standard deviations (SDs) were described for continuous variables including levels of biomedical markers within each group or each lipid abnormality class. Means and confidence interval (CI) were used for evaluating the prevalence of dyslipidemias within group/class. ANOVA was used to analyze multiple comparisons. Logistic regression analysis was applied for the relationship between the independent variables (e.g., age and proinflammatory cytokines, TBARS, and IAC) and the response variables (e.g., lipid abnormality). Pearson correlation coefficients were calculated for the association between biomedical marker parameters and lipid abnormalities. A two-tailed *p*-value of <0.05 was considered to be significant.

## Results

As shown in **Table 1**, our participant sample was composed of 1,397 adults living in the east coast, Fujian, China, whose baseline ages ranged from 18 to 83, 50.08% men and 49.92% women, and the mean age was 40.6 ± 13.8 years in men and 42.9 ± 12.9 years in women. The consecutive 3-year visits (from 2017 to 2019) yielded demographic characteristics and general socioeconomic indicators among them.

To probe the normality of the samples, we applied the Anderson–Darling test to delineate three probability plots through the overall parameters, time (years), and sex, respectively. The result showed that the data followed a normal distribution. **Table 2** indicates the levels of serum lipid profile in the consecutive years. Higher serum levels of total cholesterol, LDL-C, and triglycerides were significantly associated with aging among men and women. No significant change was observed in the 3 years within each age group.

**Table 2:** Means and standard deviations of the serum levels of cholesterol, LDL cholesterol, HDL cholesterol, and triglycerides among adults in East Coast, Fujian, China.

Gender	Group (years)	Serum Cholesterol (mg/dl)*									Serum Triglycerides (mg/dl)*		
		Non-HDL-C			LDL-C			HDL-C					
		1st year (2017)	Follow-up I (2018)	Follow-up II (2019)	1st year (2017)	Follow-up I (2018)	Follow-up II (2019)	1st year (2017)	Follow-up I (2018)	Follow-up II (2019)	1st year (2017)	Follow-up I (2018)	Follow-up II (2019)
		X ± SD	X ± SD	X ± SD	X ± SD	X ± SD	X ± SD	X ± SD	X ± SD	X ± SD	X ± SD	X ± SD	X ± SD
**Male**													
**Total**	18–29	142.0 ± 22.4	144.9 ± 26.1	145.3 ± 22.5	108.8 ± 20.8	109.4 ± 19.1	110.3 ± 20.7	57.5 ± 9.6	57.2 ± 10.1	56.8 ± 7.9	190.9 ± 20.6	191.1 ± 20.1	194.1 ± 27.1
	30–39	144.9 ± 21.9	145.8 ± 23.8	150.9 ± 23.4	110.1 ± 20.3	110.6 ± 19.9	112.9 ± 20.6	56.7 ± 8.8	56.7 ± 9.4	57.0 ± 7.4	191.6 ± 21.2	193.0 ± 22.6	195.4 ± 21.4
	40–49	153.3 ± 23.5	155.7 ± 22.6	157.3 ± 24.3	111.5 ± 20.3	113.2 ± 20.1	117.4 ± 21.1	52.1 ± 9.0	52.0 ± 9.9	51.9 ± 8.8	194.1 ± 22.7	196.7 ± 20.6	200.5 ± 28.8
	50–59	156.4 ± 21.8	159.4 ± 25.5	163.1 ± 20.9	121.9 ± 21.3	124.3 ± 20.3	127.9 ± 20.3	53.3 ± 7.8	54.0 ± 8.7	53.0 ± 9.9	188.7 ± 23.3	191.9 ± 23.6	195.4 ± 25.5
	60–69	157.2 ± 23.4	160.9 ± 24.5	163.5 ± 22.5	132.0 ± 20.7	133.0 ± 20.1	136.6 ± 19.5	52.9 ± 8.9	53.0 ± 8.5	52.4 ± 8.3	168.3 ± 25.6	171.9 ± 22.5	175.0 ± 20.4
	≥70	152.6 ± 25.2	155.9 ± 26.8	162.8 ± 21.1	135.5 ± 20.1	137.1 ± 19.2	141.0 ± 18.8	58.6 ± 9.1	58.0 ± 8.3	55.5 ± 9.5	158.0 ± 22.1	162.0 ± 22.9	165.1 ± 21.1
		153.8 ± 20.8	156.8 ± 21.4	160.7 ± 22.7	123.3 ± 19.9	123.7 ± 22.7	134.6 ± 21.6	55.4 ± 7.7	54.5 ± 8.5	53.9 ± 7.8	190.2 ± 24.1	194.6 ± 23.3	196.6 ± 22.8
*p*-values for linear trend		<0.001	<0.001	<0.001	<0.001	<0.001	<0.001	0.09	0.06	0.08	<0.001	<0.001	<0.001
**Female**													
**Total**	18–29	130.1± 22.8	130.2 ± 24.1	132.3 ± 24.6	109.2 ± 19.8	111.3 ± 19.3	112.5 ± 20.5	60.6 ± 6.6	60.3 ± 8.2	59.5 ± 8.9	159.8 ± 20.1	160.7 ± 26.5	165.4 ± 23.8
30–39	131.0 ± 19.9	131.7 ± 24.4	135.4 ± 23.6	109.4 ± 18.5	111.6 ± 17.7	112.6 ± 18.7	60.6 ± 7.1	60.2 ± 8.6	59.2 ± 9.9	160.1 ± 23.5	161.4 ± 26.6	166.6 ± 25.7
40–49	134.0 ± 22.6	135.9 ± 21.1	139.4 ± 22.6	113.4 ± 23.3	116.2 ± 18.6	122.9 ± 20.3	61.1 ± 7.4	60.1 ± 7.6	60.3 ± 7.7	160.5 ± 23.5	162.4 ± 25.5	167.0 ± 24.9
50–59	137.0 ± 22.1	142.9 ± 22.6	150.8 ± 25.8	117.4 ± 22.5	119.1 ± 16.9	126.4 ± 20.6	59.6 ± 7.9	59.3 ± 7.7	58.8 ± 8.4	167.0 ± 22.1	167.1 ± 23.8	170.7 ± 22.1
60–69	147.2 ± 24.4	149.1 ± 25.2	157.7 ± 26.1	121.2 ± 21.5	123.9 ± 22.6	130.8 ± 19.3	56.4 ± 8.5	56.1 ± 8.2	55.3 ± 10.1	177.2 ± 22.1	179.5 ± 25.8	184.1 ± 23.8
≥70	148.8 ± 24.7	153.3 ± 22.4	158.2 ± 22.6	121.2 ± 21.6	123.9 ± 23.7	131.9 ± 17.7	56.1 ± 7.4	56.4 ± 9.9	55.4 ± 9.5	175.0 ± 23.1	179.3 ± 25.4	183.8 ± 22.4
	127.0 ± 22.2	129.8 ± 23.9	135.9 ± 24.9	101.4 ± 20.5	102.9 ± 17.7	107.9 ± 19.1	51.1 ± 7.3	51.4 ± 8.1	51.3 ± 9.6	145.9 ± 22.1	147.8 ± 24.4	153.1 ± 24.6
*p*-values for linear trend		<0.001	<0.001	<0.001	<0.001	<0.001	<0.001	0.06	<0.01	0.06	<0.001	<0.001	<0.001

X, mean; SD, standard deviation. Values expressed in mg/dl [n = 15,397 (2017), 12,144 (2018), and 11,762 (2019)]. HDL indicates high-density lipoprotein; LDL, low-density lipoprotein.

*To convert from milligrams per deciliter to millimoles per liter divided by 38.67 for total, HDL, and LDL cholesterol and by 88.574 for triglycerides.

We examined the distribution of lipid abnormalities among urban adults (**Table 3**). Results showed that within each lipid profile, participants present decreases in borderline levels compared to high/very high levels over time. **Table 4** indicates the mean prevalence of various lipid profiles within the three consecutive years. A higher prevalence of dyslipidemia was significantly associated with male participants, family history, unhealthy habits (alcohol drinking and sedentary leisure activity), body weight (high waist circumference, overweight, and obesity), and health-related history (CHD, CVD, hypertension, and diabetes). The prevalence of other various cholesterols and TG was significantly higher among the participants with higher-than-average body weight (high waist circumference, overweight, and/or obese) and with a history of CVD and diabetes. The male-specific prevalence was found in high LDL cholesterol. There was a clear influence of history of smoking and hypertension on the prevalence of high triglyceride and low HDL cholesterol. There was no impact of educational level on the lipid profiles.

**Table 3:** Distribution of lipid abnormalities among adults in East Coast, Fujian, China in 2017 and 2-year follow-up.

			Prevalence, * % (95% CI)								
			1st year (2017)			Follow-up I (2018)			Follow-up II (2019)		
			Overall	Male	Female	Overall	Male	Female	Overall	Male	Female
**Sample size**			15,397	7,829	7,568	12,144	6,047	6,097	11,762	5,776	5,986
**Lipid**	**Value**	**Grading**									
**Serum Non-HDL-C (mg/dl)**	<130	Optimum	17.2 (15.3–19.1)	15.9 (14.1–19.8)	17.5 (15.9–19.5)	16.5 (15.1–18.0)	15.1 (11.4–18.8)	17.5 (15.9–19.5)	16.0 (15.1–17.0)	14.7 (12.5–16.9)	16.5 (14.9–18.2)
	130–159	Desirable	28.3 (25.5–31.1)	25.9 (23.3–29.9)	31.7 (27.5–33.5)	23.1 (25.5–31.1)	22.9 (21.3–24.6)	24.8 (22.5–27.2)	21.3 (19.5–23.1)	21.4 (20.3–22.6)	24.0 (22.0–26.1)
	160–189	Borderline	21.3 (19.6–23.2)	23.2 (21.1–25.5)	19.5 (15.7–22.3)	23.3 (19.6–23.2)	25.0 (22.6–27.5)	19.5 (15.7–22.3)	23.5 (20.6–26.4)	24.7 (22.6–26.8)	19.4 (16.7–22.2)
	190–219	High	26.2 (23.2–29.2)	28.2 (24.2–32.2)	24.2 (20.3–28.1)	29.2 (32.1–26.7)	29.6 (33.1–26.8)	28.8 (32.9–25.7)	29.0 (27.0–31.1)	28.6 (33.4–26.1)	29.3 (33.1–26.1)
	≥220	Very high	7.0 (4.1–10.0)	6.8 (4.3–9.3)	7.1 (4.1–10.1)	7.9 (10.1–6.1)	7.4 (9.3–5.5)	8.4 (11.6–5.2)	10.2 (13.1–7.3)	10.6 (13.1–7.2)	9.8 (11.7–8.3)
**Serum LDL-C (mg/dl)**	<100	Optimum	20.8 (17.7–23.9)	20.7 (17.9–23.8)	20.8 (17.7–23.9)	19.6 (17.7–21.9)	19.6 (17.6–21.6)	19.5 (17.9–21.1)	18.9 (16.1–20.7)	18.5 (16.1–20.9)	19.3 (16.1–20.7)
	100–129	Desirable	28.6 (26.3–30.9)	28.8 (25.7–31.7)	28.5 (26.2–30.8)	28.5 (26.5–30.5)	28.6 (26.5–30.7)	28.4 (26.5–30.5)	26.5 (23.7–29.3)	26.4 (23.4–29.4)	26.6 (23.7–29.3)
	130–159	Borderline	24.7 (21.9–27.5)	25.0 (23.1–27.1)	24.4 (21.8–27.6)	24.6 (21.7–27.5)	24.7 (21.9–27.5)	24.6 (21.7–27.5)	23.1 (21.4–24.8)	23.1 (21.2–24.9)	23.1 (21.5–24.7)
	160–189	High	18.6 (16.4–21.0)	18.7 (17.2–21.0)	18.5 (15.1–21.8)	18.6 (17.6–20.6)	18.8 (16.8–20.9)	18.4 (16.0–20.8)	25.1 (23.3–27.7)	24.5 (22.3–26.7)	25.7 (23.9-27.5)
	≥190	Very high	7.3 (4.7–10.1)	6.8 (5.1–8.6)	7.8 (6.6–9.1)	8.7 (7.5–9.9)	8.3 (6.5–9.9)	9.1 (7.3–11.0)	6.4 (4.8–8.2)	7.5 (6.1–9.0)	5.3 (3.1-7.7)
**Serum HDL-C (mg/dl)**	≥60	High	16.5 (14.2–18.8)	16.1 (13.7–18.5)	16.9 (13.9–19.9)	16.2 (14.0–18.4)	15.9 (14.0–17.8)	17.5 (14.1–20.9)	15.0 (13.7–16.3)	14.5 (13.2–15.8)	15.5 (13.0–18.0)
	40–59	Borderline	58.2 (56.1–60.3)	57.8 (55.8–59.8)	58.6 (55.2–61.0)	58.1 (55.9–60.3)	58.0 (55.2–60.8)	58.2 (55.4–61.0)	57.3 (55.3–59.3)	56.9 (55.0–57.8)	57.7 (56.1–59.3)
	<40	Low	25.3 (23.7–27.1)	26.1 (23.7–28.7)	24.5 (21.9–27.1)	25.7 (22.9–28.6)	26.1 (24.3–28.1)	25.3 (22.0–28.8)	27.7 (25.4–29.8)	28.6 (26.4–30.8)	26.8 (24.8-28.9)
**Serum TG (mg/dl)**	<150	Optimum	40.7 (38.1–43.3)	40.5 (38.0–43.0)	40.9 (38.1–43.7)	40.0 (38.0–42.0)	39.9 (38.0–42.0)	40.1 (38.0–42.2)	39.0 (37.7–40.3)	38.7 (37.7–40.3)	39.3 (37.2–41.4)
	150–199	Borderline	31.4 (29.8–33.0)	31.4 (29.6–33.2)	31.3 (29.5–33.1)	31.0 (29.1–32.9)	30.9 (29.1–32.9)	31.1 (29.0–33.2)	30.9 (29.0–32.8)	30.8 (29.0–32.8)	31.0 (28.8–33.2)
	200–499	High	21.0 (19.5–23.4)	22.0 (20.8–23.1)	19.9 (13.3–22.1)	22.0 (18.5–25.6)	22.7 (20.8–24.6)	21.3 (19.9–23.2)	22.6 (20.9–24.1)	23.6 (21.9–25.8)	21.6 (19.7-24.2)
	≥500	Very high	6.9 (5.1–8.8)	6.1 (4.7–7.8)	7.9 (5.9–9.9)	7.0 (5.2–8.9)	6.5 (5.1–8.0)	7.5 (5.8–9.3)	7.5 (5.0–9.1)	6.9 (5.3–8.6)	8.1 (6.1-10.1)
**Total**	From Optimum/low to Borderline*		67.3 (61.9-72.7)	66.2 (62.7–69.5)	68.4 (63.2–73.6)	65.4 (61.1–69.7)	64.2 (59.5–68.9)	66.6 (61.2–72.0)	63.6 (58.1–68.5)	62.1 (57.7–66.5)	65.5 (60.3–70.7)
	High and Very high		32.7 (30.1-34.8)	33.8 (31.1–35.8)	31.6 (30.0–33.3)	34.6 (31.1–37.5)	35.8 (33.9–37.8)	33.4 (30.0–36.6)	36.4 (32.9–39.9)	37.9 (35.9–39.9)	34.5 (32.9–36.8)

*Prevalence (%) adjusted for age according to data from Chinese population estimates for the year 2017, 2018, and 2019 according to ATP III—Third Report of the Expert Panel on Detection, Evaluation, and Treatment of High Blood Cholesterol in Adults; CI, confidence interval; LDL, low-density lipoprotein; HDL, high-density lipoprotein. *p < 0.01 (χ^2^ test).

**Table 4:** Mean prevalence of dyslipidemias according to demographic and socioeconomic variables and familial history among adults in East Coast, Fujian, China.

Prevalence, * % (95% CI)					
	Dyslipidemia^#^	High total cholesterol	High LDL-C	High TG	Low HDL-C
Male	39.54 (35.87–43.21)	34.31 (30.79–37.82)	29.33 (25.89–32.77)	29.67 (26.51–32.84)	26.10 (22.97–29.56)
Female	31.97 (28.85–34.99)	28.85 (25.79–31.92)	28.97 (25.95–31.99)	28.93 (25.92–31.92)	24.54 (22.87–26.21)
*P*	0.04	0.06	0.20	0.20	0.18
Dyslipidemia in family					
Yes	44.31 (39.15–47.99)	11.14 (8.69–13.59)	11.01 (8.24–13.80)	30.61 (25.99–35.26)	20.96 (17.29–24.91)
No	32.23 (29.99–33.02)	7.46 (5.90–8.92)	7.31 (6.58–7.71)	20.52 (16.86–23.76)	17.24 (14.39–20.09)
*p*	<0.01	0.06	<0.01	0.02	0.08
Education level					
Low education	40.56 (35.21–45.92)	10.01 (7.40–12.63)	9.52 (8.41–9.65)	26.11 (22.03–26.19)	18.01 (16.02–19.99)
College/higher education	36.03 (33.01–40.05)	8.49 (5.99–10.47)	8.78 (7.99–9.58)	25.02 (21.94–28.92)	20.20 (18.06–22.39)
*p* (Linear tread)	0.10	0.13	0.18	0.09	0.21
History of smoking ^					
Yes	42.91 (37.64–46.41)	9.93 (6.91–10.99)	9.89 (7.99–9.81)	29.12 (25.21–33.03)	26.25 (23.01–29.50)
No	33.69 (29.41–38.69)	8.61 (6.36–10.86)	8.42(7.11–9.14)	22.03 (18.96–25.02)	11.99 (9.98–13.99)
*p*	0.06	0.25	0.08	0.03	0.01
Alcohol drinking†					
Yes	42.08 (38.87–45.29)	9.84 (7.97–11.71)	10.07 (7.91–8.99)	30.76 (26.99–34.53)	27.59 (23.86–30.93)
No	34.45 (30.21–38.70)	8.71 (5.99–11.48)	8.24 (7.61–9.87)	20.42 (17.82–23.02)	10.65 (9.02–12.29)
*p*	0.04	0.13	0.07	0.03	0.01
Overweight§					
Yes	46.57 (39.86–53.28)	10.82 (8.89–12.25)	10.54 (8.95–12.13)	30.99 (27.98–34.99)	22.19 (18.97–25.39)
No	29.95 (25.03–34.97)	7.75 (5.90–9.61)	7.77 (6.59–8.91)	20.15 (17.96–22.34)	16.07 (14.76–17.37)
*p*	<0.001	<0.001	0.01	<0.001	<0.01
Obesity					
Yes	53.22 (41.98–64.57)	12.12 (10.01–14.23)	12.14 (10.97–13.35)	31.35 (28.99–33.31)	21.25 (18.89–23.66)
No	23.31 (21.44–25.23)	6.44 (5.01–7.87)	6.17 (5.49–6.85)	19.77 (17.91–21.63)	17.05 (16.21–17.89)
*p*	<0.001	<0.001	<0.001	<0.001	0.01
Waist circumference ¶					
High	49.77 (45.03–53.57)	12.38 (10.25–14.54)	12.39 (11.03–14.60)	29.75 (26.11–34.97)	23.91 (18.99–28.04)
Normal	26.83 (22.86–30.81)	6.23 (5.15–7.31)	5.94 (5.11–7.03)	21.43 (18.76–24.88)	14.36 (11.21–17.11)
*p*	<0.001	<0.001	<0.001	<0.001	0.01
Lifestyle					
Regular physical activity ~	29.84 (24.59–35.01)	7.01 (5.14–8.88)	7.11 (5.23–8.98)	21.05 (19.76–22.74)	17.42 (14.95–19.86)
Sedentary Leisure	46.69 (41.86–51.53)	11.54 (8.99–14.09)	11.25 (9.20–13.02)	30.96 (15.78–19.14)	20.81 (17.65–23.99)
*p*	<0.001	0.01	0.01	0.01	0.09
History of CHD					
Yes	40.71 (38.51–43.01)	9.28 (7.21–11.26)	10.11 (8.84–11.51)	27.39 (24.94–29.84)	17.25 (14.21–19.32)
No	35.82 (33.58–38.06)	9.23 (7.75–10.72)	8.14 (7.01–9.27)	23.72 (20.53–26.92)	20.98 (17.99–24.22)
*p*	0.01	0.12	0.06	0.10	0.10
History of CVD					
Yes	40.85 (38.22–43.48)	11.96 (9.15–15.81)	11.71 (9.02–14.41)	29.85 (24.93–35.08)	23.98 (18.90–29.10)
No	35.71 (32.29–38.21)	6.53 (4.81–8.25)	7.50 (6.01–8.99)	21.31 (19.65–23.65)	14.22 (11.13–17.04)
*p*	0.01	<0.001	0.01	0.01	0.01
Hypertension					
Yes	44.87 (41.02–48.67)	11.77 (9.01–14.61)	8.61 (7.22–9.98)	32.85 (29.40–35.73)	24.77 (19.99–29.67)
No	31.73 (28.83–34.71)	6.79 (5.02–8.68)	9.66 (7.86–10.42)	18.34 (16.30–20.80)	13.43 (10.81–16.05)
*p*	<0.001	0.01	0.15	<0.001	<0.01
Diabetes Mellitus					
Yes	46.23 (41.99–50.47)	13.74 (11.09–16.54)	12.73 (10.04–15.23)	31.15 (28.02–34.29)	25.51 (21.34–29.69)
No	30.27 (27.82–32.73)	4.83 (3.99–5.77)	5.53 (4.02–7.04)	20.03 (16.44–23.64)	12.72 (9.69–15.79)
*p*	<0.001	<0.001	<0.001	<0.001	<0.001

*Mean percentages (95% confidence interval) were shown and based on the overall values within the 3 years. CVD indicates cardiovascular disease; CHD, coronary heart disease; BP, blood pressure; LDL, low-density lipoprotein; and HDL, high-density lipoprotein.

#Dyslipidemia was defined as total cholesterol 240 mg/dl, and/or triglyceride ≥160 mg/dl, and/or LDL cholesterol 160 mg/dl, and/or HDL cholesterol 40 mg/dl, and/or use of lipid-lowering medications.

^Smoking was defined as current daily smoking or quit less than 5 years; Non-smoking is defined as never smoking cigarettes daily or quit smoking more than 5 years.

†Alcohol drinking was defined as consumption of 30 g of alcohol per week for 1 year.

§Overweight was defined as a body mass index (kg/m^2^) value of 24 or more; Obesity was defined as a body mass index (kg/m^2^) value of 28 and above. High circumference is defined as a waist circumference over 85 cm for men and over 80 cm for women.

~ Defined as an exercise of moderate or vigorous activity at least for 30 min per day with 3 days per week.

**Figure 1:**
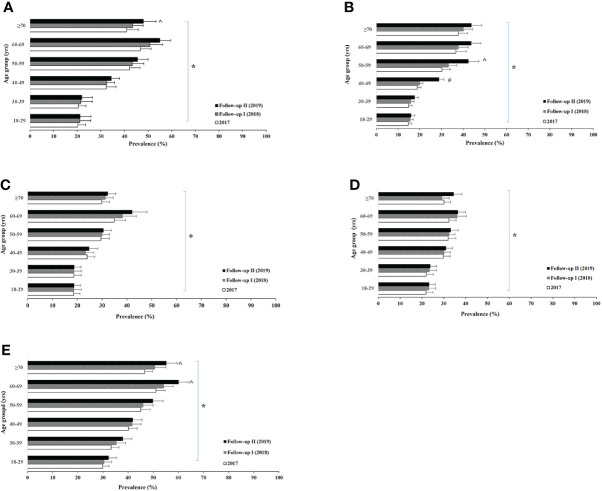
Male age-specific prevalence of high total (non-HDL) cholesterol **(A)**, high low-density lipoprotein cholesterol **(B)**, low high-density lipoprotein **(C)**, high triglyceride **(D)**, and dyslipidemias **(E)** among urban adults in East Coast, Fujian, China between 2017 and 2019. Logistic regression analysis indicated: * aging trend within each lipid abnormality **(A)** *p* < 0.005; **(B)** *p* < 0.001; **(C)** *p* < 0.001; **(D)** *p* = 0.003; **(E)** *p* < 0.001, and ^ for time trend within the age group **(B)** *p* < 0.001.

**Figure 2:**
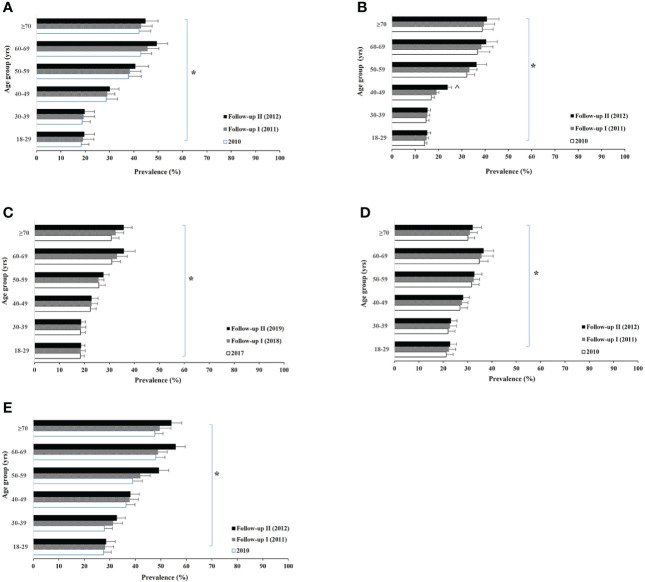
Female age-specific prevalence of high total (non-HDL) cholesterol **(A)**, high low-density lipoprotein cholesterol **(B)**, low high-density lipoprotein **(C)**, high triglyceride **(D)**, and dyslipidemias **(E)** among urban adults in East Coast, Fujian, China between 2017 and 2019. Logistic regression analysis indicated: * aging trend within each lipid abnormality **(A)** *p* < 0.01; **(B)** *p* < 0.001; **(C)** *p* < 0.001; **(D)** *p* = 0.002; **(E)** *p* < 0.001, and ^ for time trend within the age group **(B)** *p* < 0.001.

**Figures 1**, **2** show the age-specific prevalence of various lipid profiles in male and female participants, respectively. Overall, there was a significant aging trend of stable increases in prevalence of all lipid abnormalities from the young to the old group in both sexes. Maximum prevalence of dyslipidemia was in the sixth decade of life in both sexes. The prevalence of abnormal total cholesterol had a significant increase after the observed years among male participants ≥70 years of age onwards, whereas the levels were greater between 40 and 69 years in female participants. The prevalence of high LDL-C was found to significantly increase after the 3 years among the group of 40–59 years in male participants and the group of 40–49 years in female participants.

Dyslipidemia appeared much higher within the group of 60–69 years compared to others within the male population.

We examined the average levels of proinflammatory cytokines and oxidative status (TBARS and TAC) among the participants and the effects of general demographic and socioeconomic factors on the molecules (**Tables 5**, **6**). There was no difference in overall mean levels of cytokines and oxidative molecules between male and female participants. While both male and female participants had a small change of molecule levels after the observed 3 years, the majority of the participants exhibited a significant trend in favor of their age. Increases in serum IL-6, TNF-α, MCP-1, and TBARS, and reduction in serum TAC was statistically associated with the history of dyslipidemia, diabetes, and obesity.

**Table 5:** The serum levels of proinflammatory cytokines according to demographic and socioeconomic variables among adults in East Coast, Fujian, China.

Serum inflammatory marker*										
		IL-6 (pg/ml)			TNF-a (pg/ml)			MCP-1 (pg/ml)		
		1st year (2017)	Follow-up I (2018)	Follow-up II (2019)	1st year (2017)	Follow-up I (2018)	Follow-up II (2019)	1st year (2017)	Follow-up I (2018)	Follow-up II (2019)
Sex	Age (years)	X ± SD *	X ± SD	X ± SD	X ± SD	X ± SD	X ± SD	X ± SD	X ± SD	X ± SD
**Male**										
	18–29	23.6 ± 2.1	23.9 ± 2.4	23.8 ± 2.2	81.9 ± 8.2	81.8 ± 8.0	82.5 ± 8.1	86.0 ± 8.7	85.8 ± 8.4	86.1± 8.5
	30–39	23.4 ± 2.4	23.6 ± 2.2	23.9 ± 3.1	82.6 ± 8.4	82.9 ± 9.2	83.2 ± 9.2	86.2 ± 9.2	86.7± 8.6	87.1± 8.4
	40–49	23.8 ± 2.6	24.1 ± 2.6	24.4 ± 2.4	82.2 ± 8.3	83.2 ± 9.2	83.9 ± 9.1	87.1 ± 9.3	87.9 ± 9.0	88.2 ± 9.1
	50–59	23.9 ± 2.1	24.7 ± 2.4	24.6 ± 2.6	82.1 ± 8.2	82.6 ± 9.1	83.1 ± 8.9	88.2 ± 9.2	89.2 ± 9.9	90.5 ± 9.3
	60–69	24.8 ± 2.6	25.5 ± 2.5	26.0 ± 2.7	82.7 ± 8.6	83.5 ± 8.8	84.6 ± 9.0	88.1 ± 9.0	88.9 ± 9.4	89.2 ± 9.7
	≥70	25.7 ± 2.7	26.4 ± 2.4	27.9 ± 3.1	83.3 ± 8.6	85.2 ± 8.6	88.1 ± 9.0	89.5 ± 8.8	90.8 ± 10.0	91.1 ± 9.7
**Total**										
**Mean**		24.2 ± 3.0	24.6 ± 3.1	25.1 ± 3.2	82.5 ± 8.6	83.2 ± 8.4	84.2 ± 8.9	87.5 ± 8.9	88.2 ± 8.9	88.7 ± 8.8
*p* for linear trend		<0.01	<0.01	<0.01	<0.01	<0.01	<0.005	<0.01	<0.005	<0.005
**Female**										
	18–29	23.8 ± 2.3	23.5 ± 2.3	24.2 ± 2.3	81.4 ± 8.1	81.7 ± 8.2	82.1 ± 8.2	85.2 ± 8.1	85.9 ± 8.1	85.5 ± 8.7
	30–39	23.2 ± 2.4	23.9 ± 2.4	23.9 ± 2.1	82.9 ± 9.0	82.7± 9.0	82.9± 8.4	85.8 ± 8.8	86.6 ± 8.7	86.8 ± 8.8
	40–49	23.9 ± 2.1	24.7 ± 3.0	24.9 ± 2.9	82.0 ± 7.4	82.9 ± 8.4	83.3 ± 8.5	86.9 ± 8.9	86.8 ± 8.1	87.7 ± 8.8
	50–59	23.2 ± 2.3	24.4 ± 2.5	25.0 ± 3.1	82.9 ± 8.3	83.6 ± 8.9	83.9 ± 8.8	88.0 ± 9.0	88.9 ± 8.5	89.6 ± 9.1
	60–69	24.7 ± 2.5	25.0 ± 2.4	25.9 ± 2.6	82.8 ± 8.7	83.4 ± 9.1	84.1 ± 8.7	87.9 ± 8.6	88.3 ± 8.8	88.9 ± 8.8
	≥70	25.6 ± 2.7	26.8 ± 3.0	27.1 ± 2.7	83.6 ± 8.7	84.2 ± 9.1	85.6 ± 8.6	89.9 ± 8.7	92.3 ± 9.1	92.1 ± 9.1
**Total**										
**Mean**		24.1 ± 2.9	24.7 ± 2.6	25.2 ± 2.7	82.6 ± 8.8	83.1 ± 8.9	83.7 ± 8.8	87.3 ± 9.0	88.2 ± 9.1	88.3 ± 9.6
*p* for linear trend		<0.01	<0.01	<0.01	<0.01	<0.01	<0.01	<0.01	<0.005	<0.005
Dyslipidemia in family										
Yes		26.3 ± 2.9	26.3 ± 2.8	27.9 ± 3.0	94.9 ± 8.9	91.9 ± 9.2	92.9 ± 9.2	93.1 ± 9.6	93.1± 9.3	93.9 ± 9.1
No		22.1 ± 2.5	23.1 ± 2.4	24.1 ± 3.0	81.1 ± 8.1	84.1 ± 8.5	82.3 ± 8.3	87.3 ± 8.7	88.5 ± 8.9	87.1 ± 8.0
*p*		0.08	0.10	0.09	0.07	0.09	0.07	0.10	0.10	0.09
Education level										
Low education		24.9 ± 3.3	25.0 ± 3.0	26.9 ± 3.0	88.7 ± 9.2	87.7 ± 9.0	88.7 ± 9.3	89.1 ± 9.7	90.1 ± 9.1	91.1 ± 9.1
College/higher education		23.6 ± 3.2	24.8 ± 3.2	23.9 ± 3.0	83.3 ± 9.0	82.2 ± 9.0	82.3 ± 9.1	84.5 ± 9.3	82.5 ± 8.9	81.5 ± 8.8
*p*		0.12	0.15	0.10	0.10	0.10	0.10	0.10	0.10	0.10
History of smoking ^										
Yes		28.8 ± 3.6	29.1 ± 3.3	29.0 ± 3.3	89.7 ± 9.2	90.7 ± 9.2	92.7 ± 9.3	90.1 ± 9.9	88.1 ± 9.1	87.1 ± 9.11
No		23.2 ± 3.9	23.1 ± 3.0	23.9 ± 3.0	81.3 ± 9.0	81.3 ± 9.0	82.3 ± 9.1	84.5 ± 8.3	82.5 ± 7.9	83.5 ± 8.7
*p*		0.13	0.10	0.10	0.10	0.10	0.10	0.10	0.10	0.15
Alcohol drinking †										
Yes		27.3 ± 3.3	27.9 ± 3.1	27.9 ± 3.3	89.7 ± 8.7	91.7 ± 9.2	91.9 ± 9.1	90.9 ± 9.1	89.9 ± 9.1	91.5 ± 9.1
No		23.6 ± 3.4	22.9 ± 3.3	22.9 ± 3.2	80.3 ± 8.5	80.9 ± 8.6	82.3 ± 8.9	82.5 ± 8.5	81.5 ± 8.4	82.5 ± 8.7
*p*		0.11	0.09	0.09	0.08	0.07	0.10	0.09	0.11	0.10
Overweight §										
Yes		29.9 ± 3.5	30.6 ± 3.0	32.2 ± 3.9	93.7 ± 8.2	96.7 ± 8.3	98.7 ± 9.1	94.9 ± 7.8	95.7 ± 8.6	97.5 ± 9.0
No		22.0 ± 3.1	22.6 ± 3.5	23.1 ± 3.0	78.2 ± 8.1	79.1 ± 7.9	79.7 ± 8.6	79.8 ± 7.3	78.8 ± 7.7	80.3 ± 7.9
*p*		0.01	0.01	0.01	0.01	0.01	0.01	0.01	0.01	0.01
Obesity										
Yes		29.7 ± 3.3	31.4 ± 4.1	30.1 ± 3.9	95.7 ± 8.2	94.9 ± 8.2	95.5 ± 7.3	96.9 ± 8.9	97.2 ± 9.0	98.7 ± 9.0
No		22.0 ± 3.5	22.9 ± 3.2	22.8 ± 3.4	80.1 ± 8.0	80.1 ± 7.8	81.1 ± 8.1	78.0 ± 7.8	80.1 ± 8.2	82.2 ± 7.4
*p*		0.01	0.01	0.01	0.01	0.01	0.01	0.01	0.01	0.01
Waist circumference ¶										
High		28.8 ± 3.7	28.0 ± 3.0	27.9 ± 3.5	87.7 ± 9.1	89.7 ± 9.0	92.7 ± 9.3	92.1 ± 9.3	89.1 ± 9.1	94.1 ± 9.1
Normal		23.9 ± 3.2	25.8 ± 3.4	24.9 ± 3.0	83.3 ± 8.9	83.9 ± 9.0	84.3 ± 9.1	83.5 ± 8.5	83.5 ± 8.7	83.5 ± 7.9
*p*		0.08	0.11	0.10	0.09	0.10	0.07	0.06	0.10	0.06
Lifestyle										
Regular physical activity ^~^		27.1 ± 3.4	28.0 ± 3.4	26.9 ± 3.9	89.7 ± 9.2	91.7 ± 9.2	90.9 ± 9.0	92.9 ± 9.9	92.1 ± 9.10	93.6 ± 8.7
Sedentary Leisure		25.2 ± 3.5	26.1 ± 3.5	29.9 ± 3.4	80.3 ± 9.0	81.3 ± 9.0	80.8 ± 9.1	86.5 ± 10.3	85.6 ± 7.9	84.4 ± 7.9
* p*		0.12	0.20	0.25	0.15	0.15	0.15	0.15	0.15	0.15
History of CHD										
Yes		28.3 ± 3.4	29.0 ± 3.9	29.0 ± 3.9	89.7 ± 9.2	89.9 ± 9.2	92.7 ± 9.3	94.1 ± 9.9	90.1 ± 7.10	94.1 ± 9.0
No		22.8 ± 3.2	22.8 ± 3.9	23.9 ± 3.4	81.3 ± 9.0	82.3 ± 9.0	85.3 ± 9.1	83.5 ± 7.6	82.5 ± 8.0	82.3 ± 8.0
* p*		0.10	0.10	0.25	0.10	0.15	0.15	0.15	0.15	0.15
History of CVD										
Yes		29.2 ± 3.7	27.9 ± 3.9	26.8 ± 3.9	89.6 ± 9.2	88.7 ± 9.2	89.9 ± 9.3	91.8 ± 9.0	93.7 ± 7.9	92.6 ± 8.6
No		23.3 ± 3.2	23.6 ± 4.2	23.9 ± 3.4	81.3 ± 9.0	82.3 ± 9.0	82.3 ± 9.1	81.1 ± 9.0	82.5 ± 7.6	82.7 ± 8.1
* p*		0.10	0.10	0.10	0.10	0.10	0.10	0.10	0.10	0.10
Hypertension										
yes		23.6 ± 4.1	24.0 ± 3.9	24.4 ± 3.9	87.7 ± 9.2	89.7 ± 9.2	89.9 ± 9.0	91.1 ± 9.0	93.5 ± 7.7	92.4 ± 8.2
No		27.9 ± 4.3	28.5 ± 3.9	28.9 ± 3.4	83.6 ± 9.0	83.9 ± 9.0	83.2 ± 9.1	81.5 ± 10.3	82.5 ± 7.5	83.1 ± 7.4
* p*		0.25	0.25	0.15	0.15	0.15	0.15	0.15	0.10	0.15
Diabetes Mellitus										
Yes		29.5 ± 3.5	32.1 ± 4.0	32.1 ± 3.9	94.9 ± 8.2	96.8 ± 8.8	94.6 ± 8.0	96.3 ± 8.5	95.4 ± 8.10	96.9 ± 8.9
No		21.8 ± 3.0	22.3 ± 3.7	21.9 ± 3.1	79.6 ± 7.5	80.1 ± 7.5	80.1 ± 8.0	80.1 ± 7.6	79.9 ± 7.7	80.1 ± 7.8
* p*		0.01	0.01	0.01	0.01	0.01	0.01	0.01	0.01	0.01

*X, mean; SD, standard deviation. Values expressed in pg/ml [n = 15,397 (2017), 12,144 (2018), and 11,762 (2019)]. IL‐6, interleukin‐6; MCP‐1, TNF‐α, tumor necrosis factor‐α; MCP‐1, monocyte chemoattractant protein‐1. CVD indicates cardiovascular disease; CHD, coronary heart disease; BP, blood pressure; LDL, low-density lipoprotein; and HDL, high-density lipoprotein. #Dyslipidemia was defined as total cholesterol 240 mg/dl, and/or triglyceride ≥160 mg/dl, and/or LDL cholesterol 160 mg/dl, and/or HDL cholesterol 40 mg/dl, and/or use of lipid-lowering medications. ^Smoking was defined as current daily smoking or quit less than 5 years; Non-smoking is defined as never smoking cigarettes daily or quit smoking more than 5 years. †Alcohol drinking was defined as consumption of 30 g of alcohol per week for 1 year. § Overweight was defined as a body mass index (kg/m^2^) value of 24 or more; Obesity was defined as a body mass index (kg/m^2^) value of 28 and above. ¶High circumference is defined as a waist circumference over 85 cm for men and over 80 cm for women. ~ Defined as an exercise of moderate or vigorous activity at least for 30 min per day with 3 days per week.

**Table 6:** The levels of serum oxidative status according to demographic and socioeconomic variables and familial history among adults in East Coast, Fujian, China in 2017 and 2-year follow-up.

Serum oxidate status*							
		TBARS (µM)			TAC (mmol DPPH/L)		
		1st year (2017)	Follow-up I (2018)	Follow-up II (2019)	1st year (2017)	Follow-up I (2018)	Follow-up II (2019)
Sex	Group (years)	X ± SD	X ± SD	X ± SD	X ± SD	X ± SD	X ± SD
**Male**							
	18–29	3.99 ± 0.34	3.76 ± 0.33	3.95 ± 0.41	0.32 ± 0.05	0.33 ± 0.05	0.33 ± 0.03
	30–39	3.93 ± 0.34	3.98 ± 0.35	3.96 ± 0.40	0.32 ± 0.06	0.30 ± 0.6	0.32 ± 0.05
	40–49	4.02 ± 0.43	4.10 ± 0.41	4.09 ± 0.52	0.36 ± 0.05	0.37 ± 0.6	0.36 ± 0.06
	50–59	4.12 ± 0.58	4.19 ± 0.44	4.14 ± 0.55	0.39 ± 0.05	0.39 ± 0.06	0.40 ± 0.07
	60–69	4.19 ± 0.60	4.22 ± 0.55	4.29 ± 0.56	0.37 ± 0.05	0.38 ± 0.04	0.38 ± 0.06
	≥70	4.38 ± 0.60	4.48 ± 0.62	4.49 ± 0.56	0.33 ± 0.04	0.34 ± 0.05	0.33 ± 0.05
**Total**							
**Mean**		4.11 ± 0.44	4.12 ± 0.48	4.15 ± 0.47	0.35 ± 0.04	0.35 ± 0.04	0.35 ± 0.04
P values for linear trend		<0.01	<0.01	<0.01	<0.01	<0.01	<0.01
**Female**							
	18–29	3.76 ± 0.40	3.72 ± 0.37	3.79 ± 0.35	0.33 ± 0.03	0.33 ± 0.04	0.34 ± 0.03
	30–39	3.91 ± 0.42	3.99 ± 0.41	3.94 ± 0.38	0.35 ± 0.6	0.33 ± 0.04	0.32 ± 0.04
	40–49	4.04 ± 0.43	4.01 ± 0.42	4.09 ± 0.51	0.38 ± 0.07	0.39 ± 0.06	0.37 ± 0.04
	50–59	4.10 ± 0.43	4.13 ± 0.45	4.12 ± 0.47	0.40 ± 0.06	0.40 ± 0.06	0.39 ± 0.08
	60–69	4.14 ± 0.45	4.18 ± 0.41	4.22 ± 0.48	0.38 ± 0.06	0.36 ± 0.05	0.37 ± 0.04
	≥70	4.39 ± 0.46	4.49 ± 0.50	4.44 ± 0.48	0.34 ± 0.04	0.31 ± 0.05	0.32 ± 0.03
**Total**							
**Mean**		4.06 ± 0.48	4.09 ± 0.43	4.10 ± 0.47	0.36 ± 0.05	0.35 ± 0.06	0.35 ± 0.06
*p*-values for linear trend		<0.01	<0.01	<0.005	<0.01	<0.01	<0.01
Dyslipidemia in family							
Yes		4.78 ± 0.71	4.79 ± 0.69	4.81 ± 0.71	0.31 ± 0.05	0.31 ± 0.06	0.29 ± 0.05
No		3.39 ± 0.60	3.52 ± 0.65	3.63 ± 0.66	0.40 ± 0.05	0.42 ± 0.06	0.39 ± 0.06
* p*		0.03	0.06	0.07	0.06	0.06	0.05
Education level							
Low education		4.41 ± 0.62	4.28 ± 0.66	4.35 ± 0.62	0.33 ± 0.06	0.30 ± 0.06	0.31 ± 0.06
College/higher education		3.76 ± 0.69	3.93 ± 0.66	3.90 ± 0.63	0.38 ± 0.05	0.40 ± 0.06	0.39 ± 0.06
* p*		0.10	0.20	0.25	0.25	0.06	0.25
History of smoking ^							
Yes		4.78 ± 0.68	4.81 ± 0.69	4.82 ± 0.69	0.30 ± 0.06	0.28 ± 0.06	0.28 ± 0.06
No		3.39 ± 0.69	3.40 ± 0.64	3.42 ± 0.70	0.42 ± 0.06	0.42 ± 0.06	0.41 ± 0.06
* p*		0.01	0.01	0.04	0.05	0.04	0.04
Alcohol drinking †							
Yes		4.76 ± 0.69	4.79 ± 0.65	4.80 ± 0.71	0.32 ± 0.06	0.29 ± 0.05	0.28 ± 0.05
No		3.31 ± 0.68	3.42 ± 0.63	3.35 ± 0.70	0.39 ± 0.07	0.41 ± 0.06	0.42 ± 0.07
* p*		0.04	0.02	0.04	0.10	0.04	0.01
Overweight §							
Yes		4.61 ± 0.69	4.76 ± 0.71	4.79 ± 0.70	0.33 ± 0.06	0.29 ± 0.06	0.29 ± 0.05
No		3.56 ± 0.63	3.45 ± 0.69	3.36 ± 0.65	0.38 ± 0.06	0.41 ± 0.06	0.41 ± 0.07
* p*		0.08	0.04	0.05	0.20	0.07	0.07
Obesity							
Yes		4.69 ± 0.69	4.79 ± 0.70	4.81 ± 0.73	0.31 ± 0.05	0.29 ± 0.05	0.29 ± 0.06
No		3.48 ± 0.66	3.42 ± 0.66	3.34 ± 0.66	0.40 ± 0.07	0.41 ± 0.06	0.42 ± 0.06
		0.06	0.04	0.03	0.09	0.04	0.04
Waist circumference ¶							
High		4.51 ± 0.63	4.67 ± 0.70	4.57 ± 0.70	0.32 ± 0.05	0.32 ± 0.05	0.31 ± 0.06
Normal		3.66 ± 0.70	3.54 ± 0.65	3.38 ± 0.60	0.39 ± 0.07	0.38 ± 0.06	0.39 ± 0.06
* p*		0.09	0.07	0.05	0.15	0.15	0.10
Lifestyle							
Regular physical activity ~		4.54 ± 0.70	4.58 ± 0.70	4.49 ± 0.71	0.33 ± 0.05	0.38 ± 0.05	0.39 ± 0.06
Sedentary Leisure		3.63 ± 0.62	3.63 ± 0.69	3.66 ± 0.60	0.38 ± 0.09	0.32 ± 0.06	0.31 ± 0.06
* p*		0.10	0.15	0.15	0.20	0.25	0.15
History of CHD							
Yes		4.62 ± 0.69	4.55 ± 0.69	4.54 ± 0.69	0.32 ± 0.05	0.32 ± 0.05	0.31 ± 0.05
No		3.55 ± 0.67	3.66 ± 0.69	3.61 ± 0.61	0.39 ± 0.07	0.38 ± 0.06	0.39 ± 0.06
* p*		0.20	0.20	0.10	0.10	0.10	0.05
History of CVD							
Yes		4.63 ± 0.65	4.54 ± 0.69	4.53 ± 0.68	0.32 ± 0.04	0.32 ± 0.05	0.32 ± 0.05
No		3.54 ± 0.68	3.67 ± 0.64	3.62 ± 0.60	0.39 ± 0.07	0.38 ± 0.06	0.39 ± 0.06
* p*		0.20	0.20	0.15	0.10	0.10	0.07
Hypertension							
Yes		4.73 ± 0.68	4.61 ± 0.68	4.49 ± 0.63	0.33 ± 0.05	0.32 ± 0.05	0.32 ± 0.05
No		3.44 ± 0.66	3.60 ± 0.69	3.66 ± 0.60	0.38 ± 0.06	0.38 ± 0.06	0.39 ± 0.07
* p*		0.09	0.12	0.10	0.15	0.10	0.09
Diabetes Mellitus							
Yes		4.75 ± 0.69	4.79 ± 0.68	4.76 ± 0.68	0.30 ± 0.05	0.29 ± 0.04	0.29 ± 0.06
No		3.42 ± 0.63	3.42 ± 0.69	3.39 ± 0.62	0.41 ± 0.07	0.41 ± 0.06	0.42 ± 0.06
* p*		0.04	0.04	0.03	0.07	0.04	0.03

*X, mean; SD, standard deviation. Values expressed in pg/ml [n = 15,397 (2017), 12,144 (2018), and 11,762 (2019)]. IL‐6, interleukin‐6; MCP‐1, monocyte chemoattractant protein‐1; TNF‐α, tumor necrosis factor‐α. CVD indicates cardiovascular disease; CHD, coronary heart disease; BP, blood pressure; LDL, low-density lipoprotein; and HDL, high-density lipoprotein. # Dyslipidemia was defined as total cholesterol 240 mg/dl, and/or triglyceride ≥160 mg/dl, and/or LDL cholesterol 160 mg/dl, and/or HDL cholesterol 40 mg/dl, and/or use of lipid-lowering medications. ^Smoking was defined as current daily smoking or quit less than 5 years; Non-smoking is defined as never smoking cigarettes daily or quit smoking more than 5 years. † Alcohol drinking was defined as consumption of 30 g of alcohol per week for 1 year. § Overweight was defined as a body mass index (kg/m^2^) value of 24 or more; Obesity was defined as a body mass index (kg/m^2^) value of 28 and above. ¶High circumference is defined as a waist circumference over 85 cm for men and over 80 cm for women. ~ Defined as an exercise of moderate or vigorous activity at least for 30 min per day with 3 days per week.

**Figure 3:**
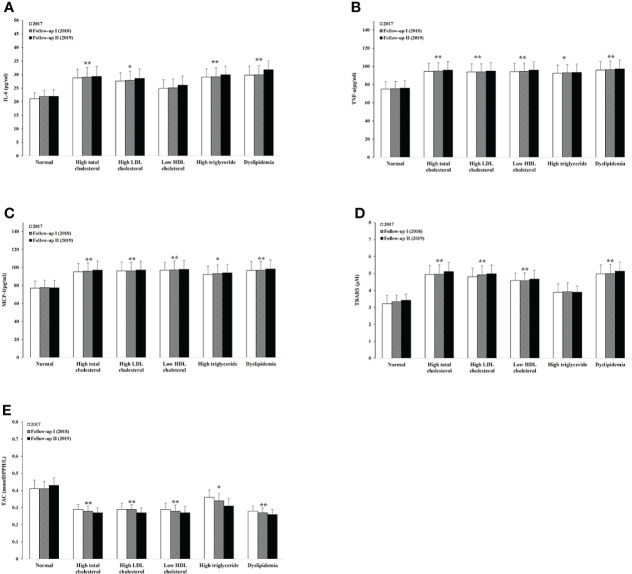
Serum levels of proinflammatory cytokines, oxidative stress, and antioxidant capacity within different lipid abnormalities among male adults. IL-6 **(A)**, TNF-β **(B)**, MCP-1 **(C)**, TBARS **(D)**, and TAC **(E)**. Logistic regression analysis indicated: *p < 0.05, **p < 0.01 overall difference vs. the level of lipid normal group.

**Figure 4:**
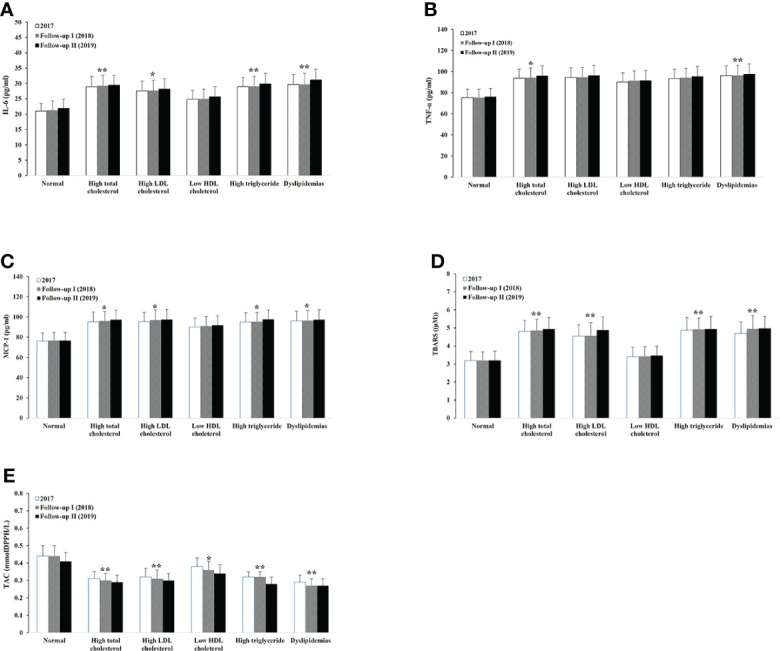
Serum levels of proinflammatory cytokines, oxidative stress, and antioxidant capacity within different lipid abnormalities among female adults. IL-6 **(A)**, TNF-β **(B)**, MCP-1 **(C)**, TBARS **(D)**, and TAC **(E)**. Logistic regression analysis indicated: *p < 0.05, **p < 0.01, overall difference vs. the level of lipid normal group.

To determine the effect of different lipid abnormalities on serum proinflammatory cytokines and oxidative status, we measured average serum levels of IL-6, TNF-α, MCP-1, TBARS, and TAC on each lipid-specific group (**Figures 3**, **4**). Compared to the normal baseline, IL-6 level was significantly higher among the groups with high total cholesterol, LDL-C, TG, and dyslipidemia. TNF-α and MCP-1 appeared to be higher for all groups with lipid abnormalities in male participants. Among female participants, TNF-α increased in groups of high total cholesterol and dyslipidemia; MCP-1 remained unchanged in the group of low HDL-C but not in other groups. Similarly, the oxidative stress representative molecule TBARS was statistically higher in groups with high total cholesterol, LDL-C, and dyslipidemia. It also increased in the male group with low HDL-C and female participants with high TG. The level of total antioxidant capacity was lower in all different abnormal lipid groups than the normal. No change of any molecular level was observed within any group during the three consecutive years.

**Table 7** demonstrates significant correlations between serum proinflammatory cytokine levels and the participant’s age and degrees of all types of dyslipidemia except low HDL-C. Similarly, such positive correlation was seen between the cytokines and obesity or diabetes but not hypertension, CVD, and CHD. TBARS level was positively correlated with age, degrees of majority of all dyslipidemia groups, with dyslipidemia in family history, and diabetes. In contrast, a negative significant correlation was found between total antioxidant capacity and age or all types of dyslipidemia.

**Table 7:** Correlation of the serum proinflammatory cytokines, oxidative status, and antioxidant capacity with relevant parameters among adults in East Coast, Fujian, China.

Correlated parameter*	Proinflammatory cytokine						Oxidative stress		TAC	
	IL-6		TNF-a		MCP-1		TBARS			
	*R*	*p*-value	*R*	*p*-value	*R*	p-value	*R*	*p*-value	*R*	*p*-value
*Demographic/socioeconomic variables*										
Age	0.380	<0.01	0.455	<0.005	0.453	<0.005	0.376	<0.01	-0.376	<0.01
Sex	0.150	0.12	0.118	0.16	0.144	0.12	0.153	0.12	0.131	0.15
Lifestyle of sedentary leisure	0.101	0.180	0.124	0.150	0.122	0.150	0.144	0.130	0.095	0.200
Waist circumference	0.163	0.090	0.168	0.090	0.166	0.090	0.196	0.060	-0.151	0.120
Obesity	0.462	<0.005	0.453	<0.005	0.464	<0.005	0.240	0.040	-0.227	0.050
CVD	0.171	0.100	0.172	0.100	0.170	0.100	0.118	0.190	-0.179	0.080
CHD	0.137	0.130	0.133	0.130	0.122	0.150	0.109	0.170	-0.170	0.100
Hypertension	0.100	0.180	0.126	0.150	0.150	0.120	0.172	0.100	-0.150	0.120
Diabetes mellitus	0.376	<0.01	0.373	<0.01	0.374	<0.01	0.277	<0.04	-0.280	<0.04
Dyslipidemia in family	0.167	0.09	0.186	0.07	0.167	0.09	0.201	0.06	0.196	0.06
*Lipid-related variables*										
Total Cholesterol	0.577	<0.001	0.558	<0.001	0.481	<0.001	0.603	<0.001	-0.548	<0.001
LDL-C	0.373	<0.01	0.395	<0.01	0.451	<0.005	0.459	<0.005	-0.520	<0.001
HDL-C	0.188	0.090	0.189	0.060	0.225	0.050	0.242	0.060	-0.451	<0.005
Triglyceride (TG)	0.644	<0.001	0.355	<0.03	0.657	<0.001	0.560	<0.001	-0.444	<0.005
Non-HDL-C	0.540	<0.005	0.541	<0.005	0.523	<0.005	0.564	<0.001	-0.471	<0.005
TG/HDL	0.379	<0.01	0.265	<0.05	0.395	<0.01	0.403	<0.01	0.268	<0.05
Dyslipidemia	0.584	<0.001	0.579	<0.001	0.587	<0.001	0.565	<0.001	-0.475	<0.002

*Presented by Pearson correlation coefficient and p-value. IL‐6, interleukin‐6; MCP‐1, monocyte chemoattractant protein‐1; TNF‐α, tumor necrosis factor‐α. TBARS, thiobarbituric acid-reactant substances; TAC, total antioxidant capacity.

## Discussion

The aim of the 3-year cross-sectional investigation was to obtain the overall profile of the prevalence of dyslipidemia, serum proinflammatory cytokines, and oxidative stress among the participants representing urban adults in east coast China. Such calculations were valued through subpopulating samples matched within categorical variables such as age, sex, family income, and education level. The finding of an elevated trend in the prevalence of dyslipidemia in the 21st century among urban populations is consistent with previous studies (20, 33, 34) in other areas of China. In our study, from 2017 to 2019, the TC levels in male participants increased by 10.4% (6.6%–14.3%). Based on clinical documentation of the association between a 10% difference in serum cholesterol and 15% difference in the risk of CHD, it is predicted that the incidence of CVD in urban east coast may increase by 17.3% in the 2020s.

The result showed an age-specific increase in the prevalence of all dyslipidemia subtypes including hypercholesterolemia, high LDL-C, hypertriglyceridemia, and low HDL-C, suggesting the possibility of more cases of CVD/CHD and diabetes as their ages advance. Such a growing public health problem was also recognized in cross-sectional surveys in Venezuela (35), Iran (36), India (37), South Africa (38), and Europe (39). Furthermore, the prevalence of dyslipidemia among middle-aged men (40 years and above) and women (30 years and above) is higher than what was previously reported in the general Chinese population, with 22.2% in men and 15.9% in women (20). Compared to some main metropolises, such as Beijing and Shanghai, urban adults residing in east coast China have a relatively equal prevalence of dyslipidemia (22). A comparably higher prevalence among middle-aged adults is different from that in the developed world (40–43) where a higher dyslipidemia occurred commonly among elders (44). In fact, our study showed a higher prevalence of dyslipidemia among urban adults with a history of CVD, CHD, or hypertension than those without it. Many factors may contribute to the trend including rapid demographic, social, and economic changes. Thus, our study suggested an important necessity of preventive interventions particularly among middle-aged adults to reduce the burden of resultant cardiovascular diseases and coronary heart diseases in east coast China.

The study showed that the five classified types of dyslipidemia had increased circulating levels of IL-6, TNF-α, and MCP-1 compared with a normal lipid group in male adults. The significance is not explained by sub-aged groups although the aging tread is partially independent of major confounders (45, 46). There were no significantly different levels of cytokines found during the three consecutive years. This evidence indicates that proinflammatory cytokine serum concentration increases with lipid abnormalities. The result that increased proinflammatory cytokines are associated with obesity is consistent with other reports (47), suggesting that body fat accumulation may induce inflammatory response through excessive intake of carbohydrates. We also stated that history of diabetes positively correlated with cytokine levels. A possible mechanism is that insulin resistance or elevated glucose is developed based on the increase in the cytokines; another possibility is that acute phase proteins such as CRP and alpha-a-acid glycoprotein induced by inflammatory response may lead to abnormal glucose metabolism in adipocytes. The evidence by some reports showed that proprotein convertase subtilisin/kexin Type 9 (PCSK9) level is positively associated with LDL-C levels in members with FH (48, 49). It possibly plays a role in inflammation balance at the atherosclerotic vascular wall by inducing the expression of inflammatory cytokines, adhesion molecules, and chemoattractants (50). One should consider measuring PCSK9 levels and vascular inflammation among the Chinese population.

Our results showed an increase in TBARS and a decrease in TAC level in most subtypes of dyslipidemia as compared to non-dyslipidemia groups, suggesting increased lipid peroxidation and decreased activity of enzymatic antioxidants in dyslipidemia among adults. Free radical production during the peroxidation of cholesterol and fatty acid is regulated by a large number of antioxidant factors. Our study shows that dyslipidemia in urban adults might be associated with a disturbance of the oxidative stress/antioxidant balance, which can be due to enhanced accumulations of free radicals and excessive antioxidant consumption. This finding supported the elevated levels of lipid hydroperoxide in the serum and is parallel to the generation of oxidative and antioxidant enzyme inactivation such as SOD and G-Px (51, 52). We also explored the correlation between diabetes and oxidative stress, indicating that hyperglycemia in diabetes contributes to oxidative stress and thus agreeing with previous studies (53, 54).

Many factors contribute to the complex changes of proinflammatory cytokines and oxidative stress among the dyslipidemias. Family history, smoking, and alcohol drinking, as well as medication compounds, can initiate the inflammatory response and oxidative stress by activating a variety of humoral and cellular mediators. In particular, excessive amounts of proinflammatory cytokines are stimulated in the early phase of inflammation and enhanced accumulations of free radicals, leading to an uncontrolled oxidative stress in the pathogenesis of lipid abnormalities.

Thus, our study suggests that proinflammatory cytokines and oxidative stress are predictive of lipid abnormalities. It is conceivable that prevention of elevated levels of cytokines and oxidative stress may delay the development of dyslipidemias, resulting in a low risk of CVD, CHD, and diabetes mortalities (55). The current study showed no sex difference in the level of proinflammatory cytokines and oxidative stress in east coast China. This fact indicates that the influence of estrogen on serum cytokines and TBARS, as well as TAC, seems to be not far-reaching.

The finding that an increase in the prevalence of dyslipidemias was associated with circumference and sedentary lifestyle may result from a considerable shift from traditional diets to high-fat diets especially those with a higher ratio of saturated to unsaturated fats in the adult population (56). The result suggests that a rapid increase in overweight/obesity likely interacts with dyslipidemia-related complications especially CHD/CVD (57). The prevalence of insufficiently physically active adults may be related to workplace conditions, the daily transport, and urban environment. The resulting positive effect on individuals’ physical inactivity is recognized as a public problem caused by industrialization, urbanization, and mechanization, as well as the high income among them, which is agreeable with other reports (58). Thus, the effective prevention and control of a high lipid profile involves a healthy lifestyle and diet (44). In terms of the prevention of the rapid increase in obesity, the WHO recommends 150 min of moderate to vigorous intensity per week in addition to usual activities. It is interesting that our study showed no significant association between serum cytokines, TBARS, and TAC, and either regular physical activity or sedentary lifestyle. A previous study indicated regular physical activity counteracts mitochondrial dysfunction and ROS generation, which present oxidative stress and inflammation in aging (59). However, some studies showed that regular exercise can induce oxidative stress and inflammation (60). The alleviative effect of regular activity on proinflammatory cytokines and oxidative stress may vary depending on the type, intensity, frequency, and duration of exercise as well as on the individual’s characteristics.

The present finding suggests an increasing burden of dyslipidemia in east coast China compared to the general Chinese population. Considering the data observed in this investigation, we suggest that proinflammation induces peroxidation of unsaturated fatty acid and oxidative modification in cholesterol due to decreased activity of enzymatic antioxidants.

### Limitations

First, although our results suggested the link between dyslipidemia and cytokines and oxidative stress, there may have been concerns as to the integrity of the current study results due to the limited ability to collect upstream social determinants of health including the long and complex causal pathways in social disadvantages, risk exposure, and health inequities. Our investigation has limited value in assessing long-term relevant risk factors such as genotype, diet intake, and energy expenditure in physical activity associated with certain changes in proinflammation indicators and oxidative stress since it was quite difficult to determine periods in exposure to risk factors of an individual’s life history on the regulation of genes controlling lipid metabolism and immune functioning. An understanding of the critical or sensitive determinants would be greatly appreciated to improve clinically meaningful results.

Second, due to the relatively modest number of sample size considering the significant population of east coast China, the relationship between dyslipidemia and proinflammation/oxidative stress generated in the study may be outright oversignified. Third, some of the data about relevant demographic and socioeconomic variables were collected and analyzed based on self-report measures; therefore, the data may be subject to bias.

Fourth, we did not have complete knowledge about potential predictors or certain definitive indicators involved in lipoprotein uptake, cholesterol synthesis, and recruitment, as well oxidative modification cholesterol such as the thickness of the inner and middle layers of the arteries, resulting in the limitations of exploring the high standard classification of dyslipidemia. Fifth, the current study did not evaluate biochemical parameters such as glutathione peroxidase, superoxide dismutase, catalase activity, and reactive oxygen species (ROS) particularly related to inflammation, oxidative stress, and antioxidants status. Recent reports indicated that high sensitivity C-reactive protein (hsCRP) concentration can reflect active systemic inflammation and independently associated with the severity of atherosclerosis (61–63). Our future study will address the role of hsCRP as an independent predictor of the risk of premature cardiovascular events within specific settings of investigation.

Finally, our study did not well analyze those who received lipid-lowering therapies (LLTs) especially non-statin lipid-lowering drugs, although only less than 6% had LLT as secondary prevention of atherosclerosis in Chinese primary care. However, it was suggested that most LLTs may have anti-inflammatory or immunomodulatory properties, either independent or not of a decrease in LDL-C (64–66).

Therefore, the noise-to-signal ratio for our current surveillance is possibly moderate, potentially leading to a biased estimate of the associations between dyslipidemia and proinflammation/oxidative stress; however, our team would like to effectively identify and accurately measure the key confounding factors in a future study but not eliminate them. Accordingly, a fulfillment of randomization with a larger sample size in distributing both known and more importantly unknown risk factors may mitigate the potential for high bias and enable the level of certainty needed to influence a clinical outcome and health policy decision.

Overall, the present study is noteworthy and has a contribution in current literature concerning the roles of proinflammatory and oxidative stress in dyslipidemia and its contribution to the various subtypes among adults. This finding is consistent with a very important public health implication: without an effective approach in prevention and control of inflammation and oxidative stress, the greater incidence of dyslipidemia can occur, and potentially, its complications such as cardiovascular disease and atherosclerosis may develop in the near future.

## Conclusion

A correlation between the prevalence of dyslipidemia and the modification of inflammation status was statistically significant. The levels of proinflammatory cytokines, oxidative stress, and antioxidant capacity in serum may reflect the contextual severity of the lipid abnormalities. The prevention and treatment of dyslipidemia and abnormal proinflammation and oxidative stress are highly recommended to be a mandatory objective to pursue in east coast China to reduce the burden of cardiovascular morbidity and mortality. Therefore, these promising results in the current study further warrant a thorough medical screening in enhanced anti-inflammatory and reduced oxidative stress to better diagnose and comprehensively treat dyslipidemia at an early stage.

## Data availability statement

The original contributions presented in the study are included in the article/Supplementary Material. Further inquiries can be directed to the corresponding author.

## Ethics statement

The studies involving human participants were reviewed and approved by Ethics committee of The First Hospital of Quanzhou affiliated to Fujian Medical University. The patients/participants provided their written informed consent to participate in this study. Written informed consent was obtained from the individual(s) for the publication of any potentially identifiable images or data included in this article.

## Author contributions

NH and SX conceived of the presented pilot study direction. NH, YL and ZY developed the project and performed the data collections. NH, CY, YH and QD verified the analytical methods. SX supervised the findings of this work. All authors discussed the results and contributed to the final manuscript.

## Acknowledgments

We thank all the participants of the study in the cities. Our thanks also go to Dr. Lijun Tang and Mr. Brett Callahan for their valuable assistance in writing review.

## Conflict of interest

The authors declare that the research was conducted in the absence of any commercial or financial relationships that could be construed as a potential conflict of interest.

## Publisher’s note

All claims expressed in this article are solely those of the authors and do not necessarily represent those of their affiliated organizations, or those of the publisher, the editors and the reviewers. Any product that may be evaluated in this article, or claim that may be made by its manufacturer, is not guaranteed or endorsed by the publisher.
